# Use of CNS medications and cognitive decline in the aged: a longitudinal population-based study

**DOI:** 10.1186/1471-2318-11-70

**Published:** 2011-11-01

**Authors:** Juha Puustinen, Janne Nurminen, Minna Löppönen, Tero Vahlberg, Raimo Isoaho, Ismo Räihä, Sirkka-Liisa Kivelä

**Affiliations:** 1 Department of Family Medicine, University of Turku, Turku, Finland; 2 Unit of Neurology, Satakunta Central Hospital, Pori, Finland; 3 Härkätie Health Centre, Lieto, Finland; 4 Turku University Central Hospital, Turku, Finland; 5 Turku Health Centre, Turku, Finland; 6 Department of Biostatistics, University of Turku, Turku, Finland; 7 Unit of Family Medicine, Turku University Central Hospital, Turku, Finland; 8 Satakunta Central Hospital, Pori, Finland

## Abstract

**Background:**

Previous studies have found associations between the use of central nervous system medication and the risk of cognitive decline in the aged. Our aim was to assess whether the use of a single central nervous system (CNS) medication and, on the other hand, the combined use of multiple CNS medications over time are related to the risk of cognitive decline in an older (≥ 65 yrs) population that is cognitively intact at baseline.

**Methods:**

We conducted a longitudinal population-based study of cognitively intact older adults. The participants were 65 years old or older and had Mini-Mental State Examination (MMSE) sum scores of 24 points or higher. The study included a 7.6-year follow-up. The use of benzodiazepines and related drugs (BZDs), antipsychotics (APs), antidepressants (ADs), opioids (Ops), anticholinergics (AChs) and antiepileptics (AEs) was determined at baseline and after a 7.6-years of the follow-up period. Cognitive functioning was used as an outcome variable measured with MMSE at baseline and at the mean follow-up of 7.6 years. Control variables were adjusted with analyses of covariance.

**Results:**

After adjusting for control variables, the use of Ops and the concomitant use of Ops and BZDs as well as the use of Ops and any CNS medication were associated with cognitive decline. The use of AChs was associated with decline in cognitive functioning only in men.

**Conclusions:**

Of all the CNS medications analyzed in this study, the use of Ops may have the greatest effect on cognitive functioning in the ageing population. Due to small sample sizes these findings cannot be generalized to the unselected ageing population. More studies are needed concerning the long-term use of CNS medications, especially their concomitant use, and their potential cognitive effects.

## Background

Epidemiological studies have produced evidence of the relationship between the use of psychotropic drugs and the risk of cognitive decline in the aged populations [1,2]. Long-term use of benzodiazepines (BZDs) has been suggested to increase the risk of cognitive decline or dementia in the aged [3-6]. It has also been suggested that the use of anticholinergic medications (AChs) may be related to the risk of cognitive deterioration in the aged [7-11].

A majority of the studies have examined the association between a single group of medications with effects on the central nervous system (CNS) and cognitive decline. Our literature search in PubMed produced only one study [2] about the concomitant use of several CNS medications and the risk of cognitive decline. The results of the study showed that the combined use of CNS medications was associated with cognitive decline in older adults who were cognitively intact at baseline [2].

We decided to test the hypothesis whether the use of one CNS medication or, in particular, the use of a combination of two or more CNS medications predict decline in cognitive functioning among older persons who are cognitively healthy at baseline.

## Methods

### Participants

The sample of this longitudinal population-based study comprised participants of the longitudinal Lieto study. The population in the first phase of the Lieto study consisted of all the residents in the municipality of Lieto born in 1926 or earlier (N = 1,283) being aged 65 or older in the study year. Of these, 1,196 (93%, 488 men and 708 women) participated [12-15]. The sample of our study comprised the participants of the first phase of the Lieto study [12-15] who scored 24 to 30 sum points in the Mini-Mental State Examination (MMSE) [16] and who were alive and participated in the second phase of the longitudinal Lieto study (N = 565; 227 men and 338 women). The first phase of the Lieto study was carried out between 1 October, 1990, and 31 December, 1991, and the second phase between 1 March, 1998, and 31 September, 1999 [12-15]. Thus, the mean follow-up time was 7.6 ± 0.5 years (range 6.4-9.1 years).

### Methods and measures

During both phases of the Lieto study all participants were interviewed about their socioeconomic background, physical and psychosocial factors, functional abilities, use of medications and health behaviour. They were also clinically examined by a health centre physician who was part of the research team (RI or ML). Clinical tests were performed by a trained research nurse. Medical records in the Härkätie Health Centre, Lieto, were used in recording previous diagnoses. Similar measures were used in both phases.

Cognitive functioning was measured during both phases with an MMSE performed by a trained nurse. The MMSE scale consists of 23 items, and the sum score ranges from 0 to 30, higher scores indicating better cognitive performance [16]. The mean change in MMSE sum scores during the follow-up was used as an outcome variable.

Information about the use of all medications prior to seven days before the interview was collected in a personal interview conducted by a trained nurse at the baseline and follow-up interviews to describe the total medication at both data collection phases. The participants had been informed to bring along their prescription forms and medications in order to confirm their current use of medication. A health centre physician from the research team (RI or ML) verified the medications from medical records. In cases where the person interviewed was unable to answer questions adequately, a close relative or caregiver provided the relevant information. If the participant was unable to visit the health centre, a trained nurse made a home visit to check the medications. All the prescribed medications (both regular and irregular) and non-prescribed medications (vitamins etc.) were taken into account.

Medications were defined by using the Anatomical Therapeutic Chemical (ATC) Classification (1996) [17]. The groups of medications defined as those with an effect on the central nervous system (CNS) and used in the analyses of this study were as follows: benzodiazepines and related drugs (BZDs) (ATC codes N05BA, N05CD, N03AE01, N05CF, A03CA, C01DA70, M05AA51, N06CA01, N02BA71), antipsychotics (APs) (ATC codes N05A, N06CA01), antidepressants (ADs) (ATC codes N06A, N06CA), opioids (Ops) (ATC codes N01AH, N02A, N02BE51, R05DA, R05FA), anticholinergic medications (AChs) (ATC codes N04A, N05AA01, N05AA02, N05AB01, N05AB02, N05AB03, N05AB04, N05AC01, N05AC02, N05AF01, N05AF03, N05AF05, N05BB01, N06AA04, N06AA06, N06AA09, N06AA12, 102AG, A03AA, A03AB, A03AX03, A03B, A03CA, A03CB31, A03DA, A03FA01, A04AD01, A04AD12, C01BA01, C01BA03, C01BA51, C01BA71, R03BB, M03B, G04BD, S01FA, R01BA01, R01BA51, R06AB01, R06AE03, R06AE53) and antiepileptic medications (AEs) (ATC code N03A) [18,19].

The usages of these groups of CNS medications were first dichotomized (regular or irregular use vs. no use). The doses of the medications were not taken into account. Eight variables describing the use of CNS medications were then formed: BZDs, APs, ADs, psychotropics (including BZDs, APs or ADs), Ops, AChs, AEs and any CNS medications (including BZDs, APs, ADs, Ops, AChs or AEs). Finally, 21 variables describing all combinations of the CNS medications were formed.

Previously known risk factors of cognitive decline [20] such as age, sex, basic education, hypertension, atrial fibrillation or flutter, diabetes mellitus, congestive heart disease and smoking at the baseline were used as control factors. Interviews were used in collecting data about basic education and current smoking. Hypertension, diabetes and congestive heart disease were defined according to clinical examination, medical history or previous diagnoses in the medical records. The diagnosis of atrial fibrillation or flutter was based on a diagnosis in the medical records or on electrocardiograms (ECG) recorded during the baseline examination.

Informed consent was obtained from all participants or their caregivers in both phases of the study. The study plans of the first and second Lieto studies were approved by the Ethical Committee of the Hospital District of Southwest Finland.

### Statistical analyses

The analyses were performed for the total population and separately for men, women and younger (65-74 yrs) and older (≥ 75 yrs) age groups. Participants who at the beginning of the follow-up were using one type of the CNS medications described in the methods and measures section were first compared with participants who did not use any of these medications at baseline and then with participants not using the medication concerned. Participants using a combination of two or more of the CNS medications were compared with participants using none of the CNS medications or their combinations.

Chi-square and Fisher exact tests were used to test differences in categorical variables between sexes, age groups and, diagnoses as well as between medication users and control groups at baseline and during the follow-up examination. The significances of changes in MMSE sum scores during the follow-up in the total population and in all subgroups were tested using the Wilcoxon signed rank test. The differences of the mean MMSE sum scores, the mean ages and the mean number of medications and the changes of mean MMSE sum scores between the groups were tested with the Mann-Whitney U test. Associations between the use of a certain group of CNS medications or the use of a combination of CNS medications and the risk of cognitive decline were first analyzed by the Mann-Whitney U test. The significances of the differences in the changes of cognitive functioning during the follow-up between the users of a certain group of CNS medications or the users of a combination of CNS medications and the corresponding control group of nonusers were first tested with the Mann-Whitney U test. After these analyses, adjusted analyses using the analysis of covariance were performed for those groups in which the associations between the use of a certain CNS medication or the concomitant use of certain CNS medications and the risk for cognitive decline were significant (p < 0.05) or tended to be significant (0.05 < p < 0.10) in these bivariate analyses. The associations of these variables (age, sex, education, hypertension, atrial fibrillation or flutter, diabetes mellitus, congestive heart disease and smoking at baseline) with the decline in MMSE sum scores were first analyzed in the total population, and only the variables that were significantly associated with decline (higher age, p < 0.001, and congestive heart disease, p = 0.002) were adjusted in the analyses of covariance.

## Results

### Background data

The majority (n = 439; 77.7%) of the participants completing the follow-up were aged 64 to74 years at baseline, and 126 (22.3%) were 75 years or older. The mean age was 70.5 years (range 64 to 89).

Of the participants, 357 (63.2%) were married, 147 (26.0%) unmarried or divorced, and 61 (10.8%) widowed. Altogether 393 participants (69.6%) lived at home with another person or other people, 169 (29.9%) lived alone, and 3 (0.5%) were institutionalized. 32 (5.7%) had received less than basic education, 484 (85.7%) basic education and 49 (8.7%) more than basic education. 523 (92.7%) were able to walk independently, 40 (7.1%) with an assisting device, and 1 (0.2%) with the help of another person. The mean number ± SD of prescribed medications used regularly was 1.9 ± 2.2, and that of medications used irregularly (as needed) was 0.6 ± 1.0.

### Use of CNS medications

At baseline, 20% of the participants used BZDs and 14% used AChs, while APs, ADs, Ops and AEs were used only by some participants (Table 1). At least one CNS medication was used by one third of the participants. The use of BZDs was more common in women than in men (25.1% vs. 13.2%, p < 0.001). The use of BZDs was more common among the older age group than among the younger one (34.1% vs. 16.4%, p < 0.001). APs were also more commonly used by older participants compared to the younger ones (8.7% vs. 3.9%, p = 0.027). Women used CNS medications more commonly than men (35.5% vs. 22.5%, p < 0.001), and the older age group more commonly than the younger one (43.7% vs. 26.4%, p < 0.001). Varying according to the medication group, 33 to 74% of participants used their medication at both the baseline and follow-up examination.

**Table 1:** Use of CNS medications.

Medication	Number of users					
	Baseline		Follow-up		Both baseline and follow-up	
	N	%	N	%	N	% of users at baseline
Opioids	9	2	43	7	3	33
Anticholinergics	78	14	104	18	37	47
Antiepileptics	7	1	7	1	3	43
Benzodiazepines or related drugs	115	20	181	32	84	73
Antipsychotics	28	5	36	6	11	39
Antidepressants	19	3	71	13	14	74
At least one CNS medication	171	30	262	46	133	78

Number (%) of participants using CNS medication at the baseline and follow-up examinations and number of participants using these medications both at baseline and during follow-up.

CNS = central nervous system

The use of combinations of the CNS medications included in the analyses was not common at baseline (Table 2). The most common combinations were BZDs and AChs (5.7% of the participants) and APs and AChs (3.9%).

**Table 2:** Use of combinations of CNS medications

Combinations of medications	Sex						Age			
	Both		Men		Women		65-74		75+	
	(N = 565)		(N = 227)		(N = 338)		(N = 439)		(N = 126)	
	N	%	N	%	N	%	N	%	N	%
Opioid **and**										
anticholinergic	1	0.2	0	0	1	0.3	1	0.2	0	0
antiepileptic	0	0	0	0	0	0	0	0	0	0
benzodiazepine or related drug	4	0.7	0	0	4	1.2	1	0.2	3	2.4
antipsychotic	2	0.4	0	0	2	0.6	2	0.5	0	0
antidepressant	1	0.2	0	0	1	0.3	1	0.2	0	0
any of the above drugs	6	1.1	0	0	6	1.8	3	0.7	3	2.3
Anticholinergic **and**										
antiepileptic	1	0.2	1	0.4	0	0	0	0	1	0.8
benzodiazepine or related drug	32	5.7	11	4.8	21	6.2	19	4.3	13	10.3
antipsychotic	22	3.9	6	2.6	16	4.7	13	3.0	9	7.1
antidepressant	9	1.6	3	1.3	6	1.8	8	1.8	1	0.8
any of the above drugs	48	8.5	15	6.6	33	9.8	31	7.1	17	13.5
Antiepileptic **and**										
antidepressant	0	0	0	0	0	0	0	0	0	0
benzodiazepine or related drug	3	0.5	3	1.3	0	0	2	4.6	1	0.8
antipsychotic	1	0.2	1	0.4	0	0	0	0	1	0.8
any of the above drugs	3	0.5	3	1.3	0	0	2	0.5	1	0.8
Benzodiazepine or related drug **and**										
antipsychotic	13	2.3	5	2.2	8	2.4	7	1.6	6	4.8
antidepressant	12	2.1	2	0.9	10	3.0	9	2.1	3	2.4
any of the above drugs	46	8.1	14	6.2	32	9.5	28	6.4	18	14.3
Antipsychotic **and**										
antidepressant	5	0.9	1	0.4	4	1.2	3	0.7	2	1.6
any of the above drugs	28	5.0	7	3.1	21	6.2	17	3.9	11	8.7
Antidepressant **and**										
any of the above drugs	19	3.4	4	1.8	15	4.4	15	3.4	4	3.2

Number and proportion of participants using a combination of CNS medications at baseline, by sex and age.

CNS = central nervous system

### Changes in cognitive functioning during follow-up

During the follow-up period, cognitive functioning declined significantly in the total population as well as in all the subgroups (Table 3).

**Table 3:** Cognitive functioning

Population	MMSE sum score		
	At baseline	After 7.6-year follow-up	p-value^1^
	(1990-1991)	(1998-1999)	
	Mean ± SD	Mean ± SD	
Total population (N = 565)	28.1 ± 1.9	26.1 ± 4.8	< 0.001
Men (N = 227)	28.3 ± 1.7	26.7 ± 3.4	< 0.001
Women (N = 338)	27.9 ± 2.0	25.7 ± 3.6	< 0.001
64-74 yrs (N = 439)	28.2 ± 1.8	26.9 ± 3.6	< 0.001
≥75 yrs (N = 126)	27.5 ± 2.0	23.3 ± 6.7	< 0.001

Cognitive functioning as measured by the Mini-Mental State Examination (MMSE) at baseline and after a 7.6-year follow-up, by sex and age.

^1^significance of difference between baseline and follow-up, Wilcoxon signed rank test

SD = standard deviation

### Background data and the use of CNS medications

The persons who used CNS medications also used more other medications than the non-users (Table 4). Compared to the group of non-users, the users of CNS medications included more women, persons living alone, persons needing tools for walking, and persons suffering from depression, hypertension or transient ischemic attacks.

**Table 4:** Background data and the use of CNS medications

	CNS medication users (N = 171)		CNS medication non-users (N = 394)		
Background variable	Mean ± SD		Mean ± SD		**p-value**^**1**^
Age	71.9 ± 5.7		69.9 ± 4.9		< 0.001
Number of all medications	4.5 ± 2.7		1.6 ± 1.9		< 0.001
Number of regularly taken medications	3.3 ± 2.5		1.3 ± 1.7		< 0.001
Number of medications taken as needed	1.2 ± 1.2		0.3 ± 0.7		< 0.001
Number of all medications excluding CNS medications	3.2 ± 2.5		1.6 ± 1.9		< 0.001
Number of regularly taken medications excluding CNS medications	2.4 ± 2.1		1.3 ± 1.7		< 0.001
Number of medications taken as needed excluding CNS medications	0.8 ± 1.0		0.3 ± 0.7		< 0.001

	N	%	N	%	p-value^2^

Sex (woman)	120	70.2	218	55.3	< 0.001
Marital status					
Married	15	8.8	46	11.7	0.076
Unmarried or divorced	101	59.1	256	65.0	
Widowed	55	32.2	92	23.4	
Place of living					
At home alone	64	37.4	105	26.6	0.007
At home with other person	105	61.4	288	73.1	
In institution or nursing home	2	1.2	1	0.3	
Education					
Less than basic	13	7.6	19	4.8	0.423
Basic	144	84.2	340	86.3	
More than basic	14	8.2	35	8.9	
Ability to walk					
Independent	148	86.5	375	95.2	< 0.001
With tools	22	12.9	18	4.6	
Needs to be assisted	0	0	1	0.3	
Diagnoses					
Depression	38	22.2	25	6.4	< 0.001
Alcohol related disease	1	0.6	5	1.3	0.673
Hypertension	59	34.5	100	25.4	0.027
Hypercholesterolemia	32	18.7	60	15.2	0.303
Diabetes mellitus (type I or II)	7	4.1	25	6.3	0.288
TIA	8	4.7	3	0.8	0.004
Cerebral infarct	1	0.6	3	0.8	1.000
Cerebral haemorrage	1	0.6	1	0.3	0.514
Cerebral trauma	2	1.2	4	1.0	1.000
Malignant tumour or cancer	5	0.3	17	4.3	0.432
HIV, lues or borreliosis	0	0	0	0	1.000
Dementia (all types)	2	1.2	0	0	0.091

Background data compared between the users and non-users of CNS medications, at baseline.

^1^significance of difference between users and non-users, Mann-Whitney U

^2^significance of difference between users and non-users, chi-square test or Fisher's exact test

CNS = central nervous system

SD = standard deviation

TIA = transient ischaemic attack

HIV = human immunodeficiency virus

### CNS medications and their combinations and cognitive change

#### One CNS medication

The users of at least one CNS medication were first compared to those using no CNS medications. The second control group consisted of non-users of the CNS medications examined. The use of any kind of a CNS medication at baseline was associated with cognitive decline in the bivariate analysis (p = 0.041), but the relationship did not remain significant after adjusting for control variables. The use of Ops at baseline was associated with cognitive decline in the older age group, and the use of AChs in men (Table 5). These relationships were observable even after adjusting for control variables.

**Table 5:** Use of CNS medication and the change in cognitive functioning

Medication	Sex	Baseline MMSE					MMSE during follow-up			Change in MMSE			
		Users		Controls			Users	Controls		Users	Controls		
		N	Mean ± SD	N	Mean ± SD	p1	Mean ± SD^2^	Mean ± SD^2^	p2	Mean ± SD	Mean ± SD	p for diff	adjusted p
Opioids^1^	M+W	9	27.7 ± 1.8	384	28.2 ± 1.8	0.276	21.2 ± 7.8	26.5 ± 4.5	0.011	-6.4 ± 7.3	-1.7 ± 4.3	0.007	0.032
Opioids^2^	M+W	9	27.7 ± 1.8	556	28.1 ± 1.9	0.372	21.2 ± 7.8	26.2 ± 4.7	0.018	-6.4 ± 7.3	-1.9 ± 4.4	0.009	0.021
Anticholinergics^2^	M	29	28.2 ± 1.8 ±	198	28.3 ± 1.7	0.938	25.0 ± 4.4	27.0 ± 3.2	0.015	-3.2 ± 4.0	-1.3 ± 2.9	0.021	0.002

Opioid **and**													
any other CNS medication^1^	M+W	6	27.3 ± 2.0	384	28.2 ± 1.8	0.020	18.8 ± 8.7	26.5 ± 4.5	0.010	-8.5 ± 8.0	-1.7 ± 4.3	0.004	0.007
Benzodiazepine or related drug **and**													
opioid^1^	M+W	4	26.5 ± 1.7	384	28.2 ± 1.8	0.052	15.8 ± 8.7	26.5 ± 4.5	0.004	-10.8 ± 9.0	-1.7 ± 4.3	0.006	0.002
opioid^1^	W	4	26.5 ± 1.7	210	28.0 ± 2.0	0.097	15.8 ± 8.7	26.0 ± 5.2	0.007	-10.8 ± 9.0	-2.0 ± 5.1	0.010	0.024

Significant associations between the use of at least one CNS medication or the use of a combination of CNS medications and change in cognitive functioning (MMSE) during the follow-up of 7.6 years (1990-1999), by sex.

M = men

W = women

MMSE = Mini-Mental State Examination

SD = standard deviation

p1 = significance of difference in MMSE at baseline between users and controls, Mann-Whitney U test

p2 = significance of difference in MMSE during follow-up examination between users and control, Mann-Whitney U test

p for diff = significance of difference of change in MMSE between users and controls, Mann-Whitney U test

adjusted p = p-value for difference of change in MMSE between users and controls adjusted for control factors, analysis of covariance

^1^control group: no medication with effects on the central nervous system

^2^control group: non-users of corresponding medications

#### Combinations of CNS medications

The combination of BZDs and Ops was associated with cognitive decline among all participants and among women. The association remained significant after adjusting for control variables. The combination of Ops and any CNS medication was associated with cognitive decline among all participants. The association remained significant after adjusting for control variables.

## Discussion

Our results show that Ops and the combined use of Ops and BZDs or any CNS medications were associated with cognitive decline. In addition, we discovered that the use of AChs was associated with the risk of cognitive decline in men.

### Strengths and limitations

The complete follow-up data were obtained for 565 participants, and the material may be considered a middle-sized cohort. The longitudinal population-based design and a high participation rate (93% of the total aged population) are major methodological strengths of this study.

Medication history and clinical background data were reliably recorded. The measure of cognitive functioning (MMSE) is a frequently used instrument for assessing global cognitive functioning. It measures general cognitive performance. The MMSE cut off point 24/23 provides a sensitivity of 69% and a specificity of 99% for dementia [21]. Only the persons who were cognitively intact at baseline were included in the study population. The diagnoses of the participants obtained from the medical records of the health centre showed that cognitive impairment was not diagnosed in any of the participants using BZDs, APs, ADs, Ops, AChs or AEs at baseline.

Many potential risk factors for cognitive decline such as age, sex, education, hypertension, atrial fibrillation or flutter, diabetes mellitus, congestive heart disease and smoking at baseline could be adjusted for as control factors. Even with adjustment, observational epidemiologic studies can only show associations between risk factors and outcome, whereas exploration of causalities requires a randomized study design. However, long-lasting randomized and controlled trials with exposure to harmful effects of medications are not possible. Due to the observational, longitudinal design and small sample sizes, these findings cannot be directly generalized to an unselected ageing population using Ops or AChs, but more studies among elderly long-term Op or ACh users with larger sample sizes are needed to confirm this association.

We adjusted the results for several potential risk factors of cognitive decline, but were unable to use some medical or other conditions (e.g., the use of alcohol) as adjusting variables. The use of alcohol was not measured during the first phase; therefore, we could not use this measure as a control variable. Studies about the use of alcohol among older Finns in the late 1980s have shown that the use diminishes with increasing age, and few older people, mainly men, are heavy users of alcohol [22].

The use of CNS medications was not common among our participants, and the concomitant use of several CNS medications was quite rare. These facts have affected the strength of the statistical tests, especially when analyzing the data by sex and age. Only baseline medication data were used in the analyses. New medications may have been prescribed and previous ones may have been given up during the follow-up period. Previous results have shown that psychotropics, especially benzodiazepines, are commonly used for years [14,15,23]. In our study, from one third to two thirds of those using a certain CNS medication at baseline used similar medication at the time of the follow-up examination. This supports the hypothesis that quite a few participants used these medications during the whole follow-up period and were real long-term users.

## Results

The use of Ops predicted cognitive decline during the long follow-up period. We have not found previous studies of possible long-term effects of Ops on cognitive functioning in the aged. Ops used by our aged population were codeine (N = 3), dextropropoxyphene (N = 2), ethylmorphine (N = 2) and dextrometorphan (N = 2). The diagnoses of the users of Ops showed that these medications were used for painful arthrotic diseases. Nobody was diagnosed to suffer from cancer pain. Despite the small number of users of these medications, a relationship was found between the use of Ops and cognitive decline suggesting that the use of Ops has a negative effect on cognitive functioning. The single use of BZDs, ADs or APs was not related to the risk of cognitive decline. These relationships are previously studied mainly for the use of BZDs, and the results are controversial [3-6,24,25]. Two small experimental randomized, controlled studies showed no cognitive decline in short-term use of APs among cognitively intact persons [26,27]. In our study, the combination of BZDs or any CNS medication and Ops was related to the risk of cognitive decline. These findings support the idea that the use of a psychotropic medication alone does not inevitably effect cognitive functioning, but combining this kind of a medication with Ops may be harmful.

The use of AChs was related to the risk of cognitive decline in men. Here our results are in concordance with the results of previous studies about the use of AChs as a risk factor for cognitive decline among unselected cognitively intact older persons [7-11]. In our population, more men than women used anticholinergic psychotropics with high CNS affinity [28], while women used more commonly urge incontinence medications targeted to have peripheral effects [28]. The blood-brain barrier permeability of different anticholinergic medications may differ depending on molecules [28], age [29] or disease states [30], which might explain why the risk of cognitive deterioration was detected only among men.

The number of persons using two or more CNS medications concomitantly was small. This possesses statistical limitations. Negative results may be caused by the rather small sample size and the small number of users. Due to a lack of statistical power, negative results do not prove that CNS medications are not related to the risk of cognitive decline. Positive results, however, show strong associations in this sample. In clinical settings, the aged using these medications should be monitored more carefully to detect the cognitive decline associated to the use of the medications.

## Conclusions

The use of anticholinergics and opioids alone and the use of opioids with benzodiazepines or any psychotropic medication or any CNS medication are associated with cognitive decline in an older cognitively intact population. However, studies with larger sample sizes and about possible pathophysiologic mechanisms are needed.

## Competing interests

This study was financially supported by the 19th February Fund of the Finnish Heart Association; the Finnish Academy; the Federation of Municipalities of Härkätie, Lieto, Finland; Turku University Hospital Grant EVO; Satakunta Hospital District Grant EVO; Finnish Association for General Practice; Uulo Arhio Foundation and Finnish Cultural Foundation. All authors of the article are independent from funders and have no conflicts of interest.

JP has lectured on further education courses for physicians, nurses and physiotherapists sponsored by Janssen-Cilag, Lundbeck and Novartis. JN has no competing interests. ML has lectured on further education courses for physicians, nurses and physiotherapists sponsored by Janssen-Cilag, Lundbeck, Novartis and Pfizer. TV, RI and IR have no competing interests. SLK has given lectures on further education courses for physicians, nurses and physiotherapists sponsored by Janssen-Cilag, Pfizer, Lundbeck, Novartis and Leiras.

The funding institutes had no role in study design, data collection, analysis, or interpretation or in the preparation of the manuscript for publication.

## Authors' contributions

JP conceptualized the study, formulated the hypothesis, designed the study, performed the literature review, analyzed the data, and drafted and revised the manuscript. JN formulated the hypothesis, designed the study, analyzed the data, and, drafted and revised the manuscript. ML collected the data (second phase), designed the study, and drafted and revised the manuscript. TV designed the study, consulted in the statistical design, and drafted and revised the manuscript. RI designed the study, collected the data (first and second phase), and drafted and revised the manuscript. IR analyzed the data, and drafted and revised the manuscript. SLK conceptualized the study, formulated the hypothesis, designed the study, supervised the data collection, analyzed the data, and drafted and revised the manuscript. All authors have read and approved the final manuscript.

## Pre-publication history

The pre-publication history for this paper can be accessed here:

http://www.biomedcentral.com/1471-2318/11/70/prepub

## References

[B1] Berg S Dellasega C The use of psychoactive medications and cognitive function in older adults J Aging Health 1996 8 1 136 149 10.1177/089826439600800107 10160568

[B2] Wright RM Roumani YF Boudreau R Newman AB Ruby CM Studenski SA Shorr RI Bauer DC Simonsick EM Hilmer SN Hanlon JT Effect of central nervous system medication use on decline in cognition in community-dwelling older adults: findings from the Health, Aging And Body Composition Study J Am Geriatr Soc 2009 57 2 243 250 10.1111/j.1532-5415.2008.02127.x 19207141 PMC2744424

[B3] Bierman EJ Comijs HC Gundy CM Sonnenberg C Jonker C Beekman AT The effect of chronic benzodiazepine use on cognitive functioning in older persons: good, bad or indifferent? Int J Geriatr Psychiatry 2007 22 12 1194 1200 10.1002/gps.1811 17407168

[B4] Paterniti S Dufouil C Alperovitch A Long-term benzodiazepine use and cognitive decline in the elderly: the Epidemiology of Vascular Aging Study J Clin Psychopharmacol 2002 22 3 285 293 10.1097/00004714-200206000-00009 12006899

[B5] Lagnaoui R Begaud B Moore N Chaslerie A Fourrier A Letenneur L Dartigues JF Moride Y Benzodiazepine use and risk of dementia: a nested case-control study J Clin Epidemiol 2002 55 3 314 318 10.1016/S0895-4356(01)00453-X 11864804

[B6] Hanlon JT Horner RD Schmader KE Fillenbaum GG Lewis IK Wall WE Landerman LR Pieper CF Blazer DG Cohen HJ Benzodiazepine use and cognitive function among community-dwelling elderly Clin Pharmacol Ther 1998 64 6 684 692 10.1016/S0009-9236(98)90059-5 9871433

[B7] Carriere I Fourrier-Reglat A Dartigues JF Rouaud O Pasquier F Ritchie K Ancelin ML Drugs with anticholinergic properties, cognitive decline, and dementia in an elderly general population: the 3-city study Arch Intern Med 2009 169 14 1317 1324 10.1001/archinternmed.2009.229 19636034 PMC2933398

[B8] Han L Agostini JV Allore HG Cumulative anticholinergic exposure is associated with poor memory and executive function in older men J Am Geriatr Soc 2008 56 12 2203 2210 10.1111/j.1532-5415.2008.02009.x 19093918 PMC3952110

[B9] Ancelin ML Artero S Portet F Dupuy AM Touchon J Ritchie K Non-degenerative mild cognitive impairment in elderly people and use of anticholinergic drugs: longitudinal cohort study Bmj 2006 332 7539 455 459 10.1136/bmj.38740.439664.DE 16452102 PMC1382539

[B10] Basu R Dodge H Stoehr GP Ganguli M Sedative-hypnotic use of diphenhydramine in a rural, older adult, community-based cohort: effects on cognition Am J Geriatr Psychiatry 2003 11 2 205 213 12611750

[B11] Bottiggi KA Salazar JC Yu L Caban-Holt AM Ryan M Mendiondo MS Schmitt FA Long-term cognitive impact of anticholinergic medications in older adults Am J Geriatr Psychiatry 2006 14 11 980 984 10.1097/01.JGP.0000224619.87681.71 17068321

[B12] Isoaho R Puolijoki H Huhti E Kivela SL Tala E Prevalence of asthma in elderly Finns J Clin Epidemiol 1994 47 10 1109 1118 10.1016/0895-4356(94)90097-3 7722544

[B13] Lopponen M Raiha I Isoaho R Vahlberg T Kivela SL Diagnosing cognitive impairment and dementia in primary health care -- a more active approach is needed Age Ageing 2003 32 6 606 612 10.1093/ageing/afg097 14600001

[B14] Linjakumpu T Hartikainen S Klaukka T Koponen H Kivela SL Isoaho R Psychotropics among the home-dwelling elderly--increasing trends Int J Geriatr Psychiatry 2002 17 9 874 883 10.1002/gps.712 12221663

[B15] Linjakumpu T Hartikainen S Klaukka T Veijola J Kivela SL Isoaho R Use of medications and polypharmacy are increasing among the elderly Journal of clinical epidemiology 2002 55 8 809 817 10.1016/S0895-4356(02)00411-0 12384196

[B16] Folstein MF Folstein SE McHugh PR "Mini-mental state". A practical method for grading the cognitive state of patients for the clinician J Psychiatr Res 1975 12 3 189 198 10.1016/0022-3956(75)90026-6 1202204

[B17] National Agency of Medicines Classification of Medicines (ATC) and Defined Daily Doses (DDD) 1996 Helsinki

[B18] Socialstyrelsen Indikatorer för utvärdering av kvaliteten i äldres läkemedelsterapi [Indicators for evaluating the quality of medication therapy in the aged] 2003 Stockholm Report No.: 2003-110-20: 74

[B19] Fick DM Cooper JW Wade WE Waller JL Maclean JR Beers MH Updating the Beers criteria for potentially inappropriate medication use in older adults: results of a US consensus panel of experts Arch Intern Med 2003 163 22 2716 2724 10.1001/archinte.163.22.2716 14662625

[B20] Qiu C De Ronchi D Fratiglioni L The epidemiology of the dementias: an update Curr Opin Psychiatry 2007 20 4 380 385 10.1097/YCO.0b013e32816ebc7b 17551353

[B21] Tangalos EG Smith GE Ivnik RJ Petersen RC Kokmen E Kurland LT Offord KP Parisi JE The Mini-Mental State Examination in general medical practice: clinical utility and acceptance Mayo Clin Proc 1996 71 9 829 837 10.4065/71.9.829 8790257

[B22] Kivela SL Nissinen A Ketola A Punsar S Puska P Karvonen M Changes in alcohol consumption during a ten-year follow-up among Finnish men aged 55-74 years Funct Neurol 1988 3 2 167 178 3402816

[B23] Puustinen J Nurminen J Kukola M Vahlberg T Laine K Kivela SL Associations between use of benzodiazepines or related drugs and health, physical abilities and cognitive function: a non-randomised clinical study in the elderly Drugs Aging 2007 24 12 1045 1059 10.2165/00002512-200724120-00007 18020536

[B24] Dealberto MJ McAvay GJ Seeman T Berkman L Psychotropic drug use and cognitive decline among older men and women Int J Geriatr Psychiatry 1997 12 5 567 574 10.1002/(SICI)1099-1166(199705)12:5<567::AID-GPS552>3.0.CO;2-V 9193967

[B25] Fastbom J Forsell Y Winblad B Benzodiazepines may have protective effects against Alzheimer disease Alzheimer Dis Assoc Disord 1998 12 1 14 17 10.1097/00002093-199803000-00002 9539405

[B26] Legangneux E McEwen J Wesnes KA Bergougnan L Miget N Canal M L'Heritier C Pinquier JL Rosenzweig P The acute effects of amisulpride (50 mg and 200 mg) and haloperidol (2 mg) on cognitive function in healthy elderly volunteers J Psychopharmacol 2000 14 2 164 171 10.1177/026988110001400206 10890311

[B27] Allain H Tessier C Bentue-Ferrer D Tarral A Le Breton S Gandon M Bouhours P Effects of risperidone on psychometric and cognitive functions in healthy elderly volunteers Psychopharmacology (Berl) 2003 165 4 419 429 10.1007/s00213-002-1272-2 12459926

[B28] Todorova A Vonderheid-Guth B Dimpfel W Effects of tolterodine, trospium chloride, and oxybutynin on the central nervous system J Clin Pharmacol 2001 41 6 636 644 10.1177/00912700122010528 11402632

[B29] Pakulski C Drobnik L Millo B Age and sex as factors modifying the function of the blood-cerebrospinal fluid barrier Med Sci Monit 2000 6 2 314 318 11208329

[B30] Starr JM Wardlaw J Ferguson K MacLullich A Deary IJ Marshall I Increased blood-brain barrier permeability in type II diabetes demonstrated by gadolinium magnetic resonance imaging J Neurol Neurosurg Psychiatry 2003 74 1 70 76 10.1136/jnnp.74.1.70 12486269 PMC1738177

